# Enhancing healthy ecosystems in northern Ghana through eco-friendly farm-based practices: insights from irrigation scheme-types

**DOI:** 10.1186/s12898-019-0254-8

**Published:** 2019-09-12

**Authors:** Caesar Agula, Franklin Nantui Mabe, Mamudu Abunga Akudugu, Saa Dittoh, Sylvester Nsobire Ayambila, Ayaga Bawah

**Affiliations:** 1 Regional Institute for Population Studies (RIPS), University of Ghana, Accra, Ghana; 2 Department of Agricultural and Resource Economics, University for Development Studies, Tamale, Ghana; 3 Institute for Interdisciplinary Research and Consultancy Services (IIRaCS), University for Development Studies, Tamale, Ghana; 4 Department of Climate Change and Food Security, University for Development Studies, Tamale, Ghana; 5 Department of Agribusiness Management and Finance, University for Development Studies, Tamale, Ghana

**Keywords:** Farm management practices, Agroecosystems, Contingent Valuation Method, Chi-square automatic interaction detector

## Abstract

**Background:**

Farming practices vary from farmer to farmer and from place to place depending on a number of factors including the agroclimatic condition, infrastructure (e.g. irrigation facilities) and management mechanisms (private versus state management). These together affect the functioning and sustainability of the ecosystems. For the sustainability of ecosystems, farmers need to employ ecosystem-based farm practices. This paper examines the ecosystem-based farm management practices (EBFMPs) in private and state-managed irrigation schemes. It also analyses the drivers of farmers’ willingness to pay for EBFMPs sustainability. The study employed mixed methods design, using both qualitative and quantitative techniques of data collection through key informant interviews, focus group discussions and semi-structured questionnaires administered to 300 households. The various EBFMPs adopted by farmers were examined and descriptively presented. The Chi-square automatic interaction detector (CHAID) and multiple linear regression were used to assess the predictors of farmers’ willingness to pay for EBFMPs to enhance the health of agroecosystems. Compost application, conservative tilling, conservation of vegetation, mulching, crop rotation, intercropping with legumes, efficient drainage systems and bunding were the EBFMPs captured in this paper.

**Results:**

Farmers in privately-managed irrigation schemes (PIS) more often apply EBFMPs compared with those in state-managed irrigation schemes (SIS). The paper also found that farmers’ willingness to pay to sustain EBFMPs for healthy ecosystems is significantly determined by the type of irrigation scheme they cultivate in (that is, PIS or SIS), their level of education, marital status and perception of soil fertility.

**Conclusions:**

Policy makers, implementers, and other stakeholders need to consider the capacity building of irrigation farmers, especially those in SIS in northern Ghana by educating them on agricultural production and ecosystem nexus to enhance the level of usage and willingness to pay for EBFMPs sustainability.

## Background

The sustenance of many livelihoods, especially in Africa ties much to the health of the ecosystems [1]. Ecosystems provide a range of services essential for human existence, which include the provision of food, climate regulation, cultural value (e.g. aesthetic and recreation), and soil nutrients cycle and formation [2, 3]. Yet, the activities of farmers in agricultural production continue to be environmentally unsustainable [4, 5]. This negatively affects the biological functioning of agroecosystems. Most farmers in Ghana aim to increase farm yields in the short-run and so are usually willing to trade-off sustainable agricultural practices for higher yields [1, 5]. This trade-off is more pronounced among younger and active farmers who have limited knowledge of the functioning of agroecosystems [5]. Research [e.g. 6, 7] further indicates that farmers’ odds for adopting sustainable agricultural practices decreases with lower educational level, a perception that the soil is fertile, leased lands, less wealth and limited access to information and credit loans [6, 8–11]. The odds for adopting sustainable farm practices, however, increases with household labour availability, livestock ownership and off-farm income [12]. If farmers’ adoption behaviour of farm practices continue to harm the environment, the ability of the agroecosystems to provide essential services to humanity will worsen [13] and this could trap future generations in poverty through high costs of agricultural production [2]. Therefore, to provide a plausible argument for policy on sustainable production, valuation of ecosystem services, a reflection of what society is willing to trade-off to sustain the natural resource base for sustainable livelihoods is crucial [14–16]. One way of achieving this is through the application of ecosystem-based farm management practices (hereafter referred to as EBFMPs).

Ecosystem-based farm management practices refer to farm-based practices that help to conserve soil fertility and improve on the general functioning of agroecosystems [17]. These are usually indigenous practices adopted by farmers, which can balance agricultural output and the functional capacity of agroecosystems. Specifically, the EBFMPs under consideration are compost application,^1^ conservative tilling,^2^ conservation of vegetation,^3^ mulching,^4^ crop rotation,^5^ intercropping with legumes,^6^ efficient drainage systems^7^ and bunding.^8^ The success or otherwise of EBFMPs depends on the magnitude of returns to investments and farmers’ willingness to pay (WTP) for the services provided [14]. Following Pascual et al. [14], if the sustainability and costs of these services are not evaluated, policy would be misleading and society, in general, would be worse off due to misallocation of resources.

The plethora of studies on sustainable farming in Ghana [e.g. 4, 18, 19] have paid little or no attention to the valuation of services provided by the EBFMPs adopted by farmers. Also, irrigation landscapes provide a lot of services useful in sustaining many livelihoods in Ghana, yet the attention given to the various irrigation scheme-types is uneven. According to Dittoh et al. [20], although attention has recently been given to private-based irrigation schemes (small scale irrigation schemes), it is not in the same magnitude as to state-managed irrigation schemes.

As such, first, this paper sought to identify and describe the types of EBFMPs that exist across different irrigation scheme-types in northern Ghana. Secondly, it sought to identify and analyse the factors that influence farmers’ willingness to pay for sustainable agroecosystems through EBFMPs. The hypothesis is that socio-economic and demographic attributes of farmers are associated with WTP for EBFMPs. The paper highlights the need to employ sustainable agricultural production practices and emphasizes the variations in EBFMPs among farmers across different irrigation scheme-types. This will offer insights for future agricultural policy and sustainable investments within the agricultural sector of Ghana and elsewhere in the developing world.

## Valuation of ecosystem-based farm management practices: empirical issues

In as much as economic valuation is important, understanding the procedural elements involved is of higher interest for a realistic and general appreciation of outcome values. This means that for EBFMPs valuation to become a strong consideration for policy formulation, it is prudent to get the true value of them [21]. There are several studies on economic valuation [e.g. 15, 16, 21–25] all of which appreciate the existence of controversies in determining the true value of most ecosystem services, especially non-marketed services. According to Gómez-Baggethun and Ruiz-Pérez [24], the general critique of ecosystems services valuation is that attempting to do so means commodifying and commercializing the services, and for ethical reasons, some things ought not to be monetized since this will grossly undervalue them. Also, Simpson [21] posits a similar argument by highlighting much on the ‘paradox of valuation’, and generally classifies valuation as one of the problematic studies in the scientific community. That is, ecosystem services valuation remains a flawed exercise because the scarcity of resource always has a role in determining the monetary value of the resource irrespective of the necessity or utility of the resource. For example, water is a fundamental necessity of human survival and yet, it is always undervalued as compared to diamond, which is highly valued just because it is a scarce commodity.

There is, however, a strong counter-argument that valuation is not the same as the commodification of ecosystem services (in whatever units) [15]. Following Costanza [15], it is a misconception to assume that valuing ecosystem services in monetary units is the same as commodifying them. The reason being that the main objective of the valuation exercise is to determine the extent to which an individual or group of people appreciate(s) the services provided by nature. This, however, can realistically be achieved through the allocation of monetary units since money has global recognition. In the viewpoint of Fisher, Bateman and Turner [23], price is simply a portion of an underlying value hence decision makers should be interested more in value than price.

From the foregoing, to measure non-marketed products, one needs to employ willingness to pay (WTP) rather than what actually has to be paid. To elicit WTP, the contingent valuation is critical as it is acknowledged by many studies [e.g. 14, 15, 23, 25] as an effective survey technique for determining the value of most non-marketed products such as EBFMPs. The Contingent Valuation Method (CVM) measures non-marketed goods by eliciting the number of people that are willing to pay and of what value for such goods. It creates a hypothetical market for the amenity such that responses can be evaluated in a manner equivalent to a behaviour observed in the markets [26]. However, it has certain limitations and advantages that need to be acknowledged. The major limitations of the technique include its subjectivity and inappropriate estimation of values when there is a dearth of knowledge by respondents. It is nevertheless considered as the most flexible technique to estimate reliable economic values when the survey design is carefully implemented [27].

## Results

### Socio-demographic profile of farmers

Table 1 shows that about 71% of the farmers are males while 29% are females. More male farmers cultivate in SIS (84%) compared with PIS (58%) and the difference is statistically significant at 1%. Approximately, 65% of the farmers that responded are married and the rest otherwise. There are more farmers who are married in SIS (≈ 73%) compared with farmers in PIS (58%) and the difference is significant at 1%. From the Table 1, it is those in their economically active age that are into irrigation farming, as the mean ages of farmers are about 38 and 45 years respectively for the SIS and PIS, and the difference in mean ages is significant at 1%. The table further suggests that the average farm size in SIS is above 1 acre (≈ 1.6 acres) relative to a little above half an acre (0.6 acres) for a counterpart in PIS. Again, Table 1 shows that the household size of farmers in PIS (≈ 6 members) is relatively larger than those cultivating in SIS (≈ 5 members), and this is statistically significant at 1%. About 45% of the farmers had no formal education, 21% for primary and JHS education apiece, and 13% for SHS education and higher. Table 1 further shows that there are more farmers with no formal education in PIS compared with farmers in SIS. It is evinced that farmers in PIS have a relatively better knowledge of the EBFMPs than their counterparts in SIS, which is also significant at 1% (Table 1). Also, a significant number of farmers (≈ 45%) in PIS had the perception that their farmlands are fertile compared with farmers in SIS (≈ 17%).

**Table 1:** Bivariate analysis of farmers’ socio-economic and demographic characteristics

Variable	Mean		Standard deviations		t-test (mean comparison) df = 298
	SIS	PIS	SIS	PIS	Pr(|T| > |t|)
Age	38.07	45.19	10.01	11.10	0.00
Household size	5.38	6.17	1.93	2.69	0.00
Irrigation farm size (acres)	1.64	0.60	1.09	0.39	0.00
Perceived knowledge of EBFMPs (indexed)	15.38	16.99	3.25	3.86	0.00

	Percentage			Pearson Chi^2^(1)	p-value (Pr)
	SIS	PIS	Total		
Sex					
Male	84.00	58.00	71.00	24.62	0.00
Female	16.00	42.00	29.00		
Marital status (dummy)					
Married	72.67	58.00	65.33	7.12	0.00
Not in union	27.33	42.00	34.67		
Perception of soil fertility					
Fertile	17.33	44.67	31.00	26.19	0.00
Less fertile	82.67	55.33	69.00		
Education (categorical)					
No formal education	36.67	52.67	44.67	8.67	0.03
Primary education	26.00	16.00	21.00		
Junior High School education	22.67	19.33	21.00		
Senior High School education and higher	14.67	12.00	13.33		

### Distribution of EBMFPs adoption

**Fig. 1:**
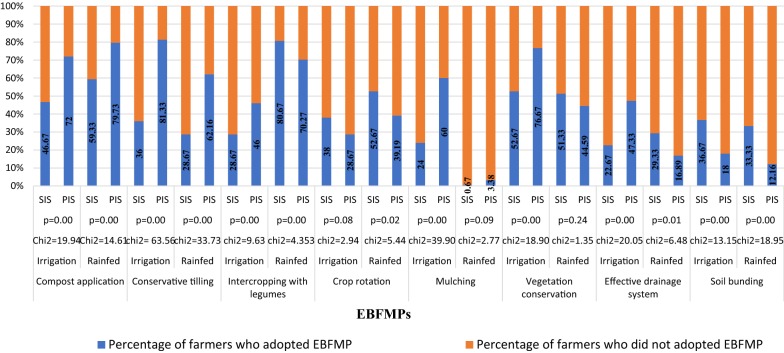
Distribution of ecosystem-based farm management practices (Source: Field survey (2016))

Figure 1 shows the level of usage of the various EBFMPs in PIS and SIS. Eight EBFMPs were identified and used by farmers in both schemes (Fig. 1). These practices are organic manure application, conservative tilling, intercropping with legumes, efficient drainage systems, conservation of vegetation, mulching, crop rotation and bunding. From the figure, there are more farmers (≈ 70%) in PIS who apply organic manure on their irrigated farms. In the SIS, a relatively lower percentage of farmers (≈ 50%) apply organic manure on their farms. The difference is statistically significant at 1%. A similar pattern is observed on rain-fed farms (Fig. 1).

Also, about 80% of farmers in PIS are using simple tools to till their irrigated farms compared with 36% for farmers in SIS, and this is significant at 1%. Similar to irrigated farms, about 62% of the farmers under PIS employ more conservative tilling practices during the raining season than their counterparts (≈ 29%) in SIS (Fig. 1). The figure also revealed that 46% of farmers in PIS inter-crop with legumes on irrigated farms while about 29% of farmers inter-crop with legumes in SIS, and the difference is significant at 1%. Unlike irrigated farms, more farmers inter-crop with legumes on rain-fed farms. The figure indicates that about 70% and 80% of farmers inter-crop with legumes in PIS and SIS respectively, and the difference is statistically significant at 1%.

Again, Fig. 1 shows that about 47% of farmers in PIS are efficient in managing water resource on irrigated farms while about 22% are efficient in SIS. On rain-fed farms, more than 70% of farmers (in both schemes) tend to have porous farm-based drainage systems. Juxtaposed with SIS, most farmers cultivating in PIS practice mulching on their irrigated farms. It was found that 60% of farmers in PIS practice mulching on their irrigated farms while 20% of farmers in SIS practice mulching (Fig. 1). Comparatively, most of the farmers (more than 95% of farmers in both schemes) do not practice mulching on rain-fed farms.

The figure further posits that about 77% of the farmers in PIS conserve the vegetation of their irrigated farms by not burning compared with 53% of farmers in SIS. The difference is statistically significant at 1%. Figure 1 also suggests that about 45% of farmers in PIS conserve their rain-fed farms vis-a-vis 51% of farmers in SIS. Another EBFMP, which is poorly adopted, is crop rotation. From the figure, about 29% of farmers in PIS practiced crop rotation on their irrigated farms while 38% of farmers practiced crop rotation in SIS. More also, the figure indicates that 18% of farmers in the PIS are practicing soil bunding on irrigated farms while about 33% of farmers in SIS practice soil bunding.

### Drivers of farmers’ WTP for EBFMPs sustainability

**Fig. 2:**
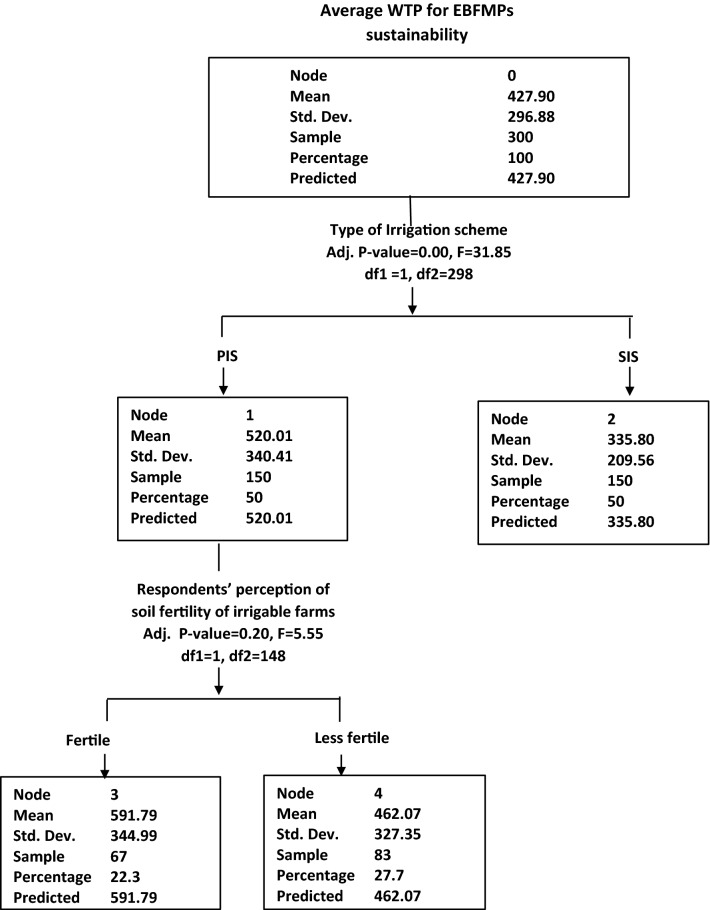
CHAID tree analysis of farmers’ willingness to pay values (Source: Field survey (2016))

Figure 2 gives a tree analysis of farmers’ willingness to pay for sustained EBFMPs through the CHAID. The monetary values obtained from the CHAID (Fig. 2) show the extent to which farmers value the services provided by the EBFMPs in maintaining the health of the agroecosystems. The CHAID tree indicates that PIS farmers are willing to pay an amount of GHS520.00 (USD108)^9^ to sustain EBFMPs compared with GHS335.00 (USD70) by the SIS farmers (Fig. 2). It further diagnosed that farmers under PIS who perceive their farmlands are fertile were more willing to pay a higher amount (GHS591.00 or USD124 equivalent) to sustain the services of the agroecosystems compared with those who perceive their farmlands as less fertile (GHS462.00 or USD97 equivalent) and this is statistically significant at 5%.

Table 2 also suggests that willingness to pay for sustained EBFMPs is determined by the type of irrigation scheme farmers are using, level of education, perception of soil fertility and marital status. Adjusting for other factors in the model, farmers in PIS are willing to pay about GHS178.00 (USD37) more for the eco-friendly practices than their counterparts in SIS, and this is significant at 1%. This outcome corroborates the CHAID analysis that farmers in PIS place more value on services provided by the EBFMPs. Another key outcome from the linear regression is the level of education. Table 2 suggests that controlling for the other variables, those who had primary education or SHS education and higher are more willing to pay to sustain EBFMPs for healthy agroecosystems than those who had no formal education, and this is statistically significant at 5%. Specifically, farmers with primary education are willing to pay about GHS97.00 (USD20) more than those who had no education. Also, farmers with SHS education and higher are willing to pay about GHS129.00 (USD27) more than those with no formal education. Holding other factors in the model constant, farmers who are married are willing to pay about GHS81.00 (USD17) more for the sustenance of EBFMPs than those who are not married, and this is significant at 5%. Lastly, farmers who perceived that their farmlands are fertile are willing to pay about GHS66.00 (USD14) more to sustain EBFMPs than those who perceived their lands are less fertile. This outcome is statistically significant at 10% and corroborates the CHAID analysis.

**Table 2:** Coefficient estimates of factors that influence WTP for eco-friendly practices

Variable	Coeff.	Std. err.
Age	1.45	1.64
Level of education		
No formal education (Ref)		
Primary education (1 = had primary education, 0 = otherwise)	97.17**	44.19
JHS education (1 = JHS, 0 = otherwise)	− 42.81	43.744
SHS education and higher (SHS and higher = 1, 0 = otherwise)	128.74**	51.41
Marital status (1 = married, 0 = not in union)	80.88**	34.65
Perceived knowledge of EBFMPs (indexed on each EBFMP importance stated)	2.14	4.66
Soil perception (1 = fertile, 0 = less fertile)	66.35*	38.28
Irrigation farm size (acres)	2.37	19.51
Type of irrigation scheme (1 = PIS, 0 = SIS)	178.31***	39.79
Number of obs = 300		
F(9, 290) = 6.93		
Prob > F = 0.00		
R-squared = 0.18		

*, **, *** Represent 10, 5 and 1% levels of significance respectively

## Discussion

The paper details the types of EBFMPs that exist across irrigation scheme-types and the level of usage. Secondly, it identified the factors that influence farmers’ WTP for sustainable eco-friendly practices. The results suggest that there is generally low level of usage of the EBFMPs identified. However, farmers in PIS adopt more of these sustainable practices than their counterparts in SIS. The reason for the disparity partly ties much to the size of the farms in PIS and SIS. It is difficult for most of the farmers in SIS to fully adopt the EBFMPs owing to the large size of their irrigable lands. This finding suggests that most of the farmers under SIS who are relatively younger prefer to trade-off nutrition and sustainability for higher incomes. On the contrary, farmers under PIS who are older prefer adopting EBFMPs for better nutrition and enhanced ecosystems. Previous studies [28–30] suggest that organic production does not only improve soil fertility, nutrition and taste, but helps curb the spread of diseases, in addition to preventing crops from easy rot. Gordon, Finlayson and Falkenmark [31] also reported that is crucial to adopt EBFMPs, especially an efficient drainage system, to promote aquatic life and ensure ecosystem resilience.

The types of crops cultivated across the schemes also account for the low adoption of the EBFMPs. The predominant crops cultivated under PIS in the dry season are leafy vegetables and other vegetables such as onions and garden eggs. These crops are relatively easier to manage compared to rice and pepper, which are cultivated on a large scale under the SIS. The type and flexibility of managing crops aid the adoption of more EBFMPs. For instance, pepper and rice production is perceived to be lucrative, as such, farmers are more reluctant to rotate such crops.

Also, farmers’ knowledge of the functioning of the various EBFMPs relates to the low usage of these eco-friendly practices. As evinced in Agula et al. [5], farmers with more insights on the biological functioning of the eco-friendly practices tend to employ more EBFMPs. Most farmers, especially in northern Ghana, focus more on yields and short-term gains with no consideration for sustainability [1, 5].

The CHAID tree highlights that farmers under PIS do not only adopt more of the EBFMPs but also, are willing to pay more for these eco-friendly practices than their counterparts in the SIS. This finding is corroborated by the multiple linear regression, which controls for other factors. Most of the farmers in PIS have in-depth knowledge about the functioning of the EBFMPs, and this explains why they are willing to pay more for their sustainability. The multiple regression results also suggest that farmers who perceive their farmlands to be fertile are willing to pay more for EBFMPs for enhanced ecosystems. The CHAID tree further diagnosed that farmers who are in PIS and perceived that their farmlands are fertile were those willing to pay the highest amount for the services provided by the EBFMPs. One would expect farmers who perceived that their farmlands are less fertile to have experienced the effect and be willing to pay more for the services of the EBFMPs. However, farmers with less fertile soils rather go in for more inorganic fertilizers and chemicals to improve the fertility of their farms instead of using EBFMPs.

Again, the multiple linear regression results suggest that formal education is associated with a high willingness to pay value for EBFMPs sustainability. Specifically, farmers with primary or SHS/Vocational/Technical education place a high value on the services provided by the EBFMPs and hence, willing to pay for these practices. This outcome is consistent with Amusa et al. [32] study which reported that educated farmers are more willing to pay for agronomic soil conservation practices. Lastly, the findings from the multiple regression results suggest that farmers who are married value the services provided by the EBFMPs more than those not married and are willing to pay more to sustain the services. This outcome is inconsistent with the finding of Aydogdu and Bilgic [33] who reported a negative relationship between married farmers and willingness to pay for efficient irrigation and sustainable usage of resources. Their reason is, married farmers would relatively have larger household sizes that possibly constrain them financially to be able to pay more for the usage of such resources.

## Conclusions

This paper sought to assess how healthy ecosystem services could be enhanced through ecosystem-based farm management practices (EBFMPs) that exist among different types of irrigation schemes for sustainable agricultural production in Ghana, taking the Kassena-Nankana area as a case study. Specifically, it sought to examine the types of EBMFPs that exist among private and state-managed irrigation schemes and to analyse the determinants of farmers’ willingness to pay for EBFMPs sustainability. It was observed that farmers under PIS adopted more of the EBFMPs than their counterparts under the SIS. Furthermore, farmers’ willingness to pay is associated with the type of irrigation scheme they cultivate in, their level of education, perception of soil fertility and marital status. Therefore, to enhance farmers’ willingness to pay for EBFMPs sustainability, the aforementioned factors should be considered. Also, there is the need for policy makers and implementers to relook at the allocation of investments among irrigation schemes to ensure that PIS have the needed support for sustained promotion of healthy ecosystems. More also, there is the need to build the capacity of farmers, especially those in SIS by educating them on agricultural production and agroecosystem nexus to enhance adoption of more EBFMPs.

## Methods

We used the methodology previously described by Agula et al. [1, 5] and Agula [17]. Details for the methodology are provided in the proceeding sub-headings.

### Study setting and sampling process

**Fig. 3:**
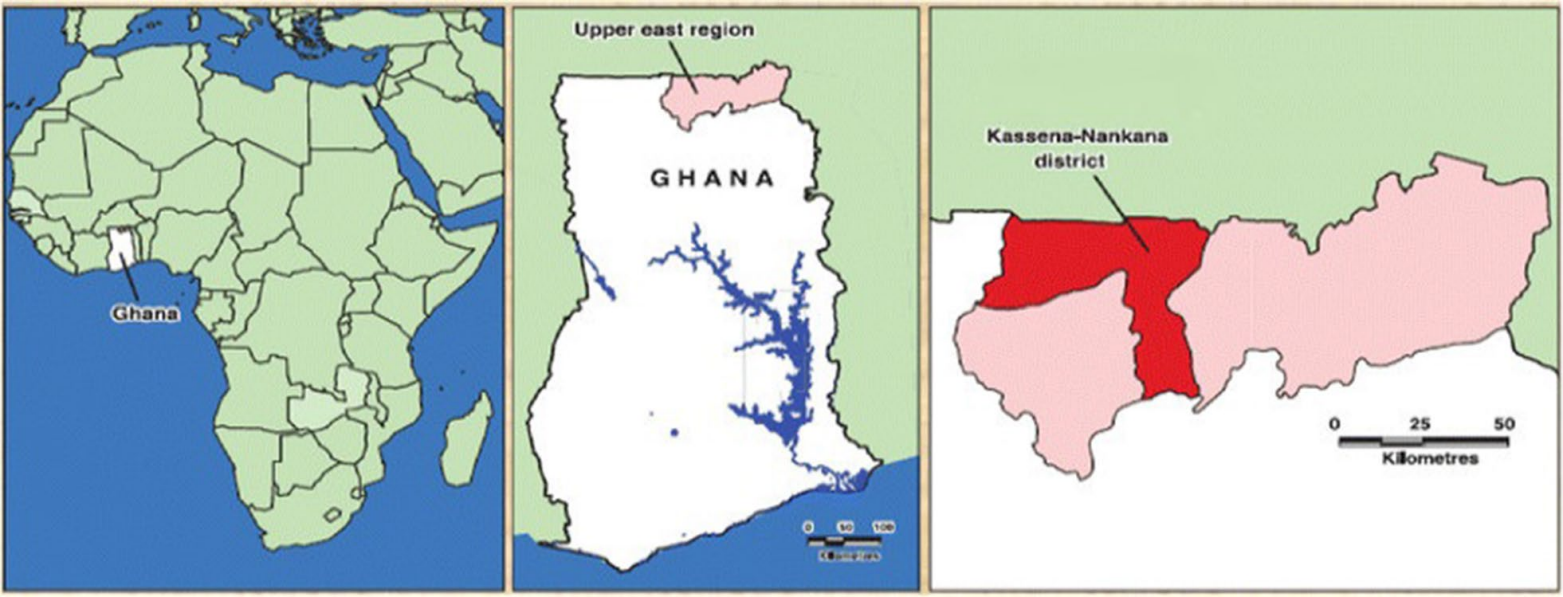
A map of Kassena-Nankana area in upper east region of Ghana (Source: [35])

The study was carried out in the Upper East Region of Ghana where the ecosystems are most fragile. This makes the need for EBFMPs adoption imperative to ensure sustainability in agricultural production. Specifically, the study was conducted in the Kassena-Nankana area (Kassena-Nankana West and Kassena-Nankana East Districts), because of the presence of small and large-scale irrigation schemes that play key roles in sustaining livelihoods of people in the area and beyond. The study districts (Fig. 3) fall within the Sudan-Savannah agroecology and have a total population of about 181,000 people, of which about 61% are in the Kassena-Nankana East District and the remaining 39% in the Kassena-Nankana West District [34]. The predominant economic activity in the area is farming with about 69% of the total population working in agriculture [28].

A three-stage sampling technique was used to select study communities and households. The first stage was the division of the study area into irrigable and non-irrigable communities and then the irrigable communities into strata of private-managed and state-managed irrigation schemes. Three communities were randomly selected from each stratum—PIS and SIS. Private-managed irrigation schemes (PIS) within the context of this paper are small-scale, where farmers access their water from a local community dam and manage their own irrigation activities. State-managed irrigation schemes (SIS), on the other hand, are large irrigation schemes in which farmers have access to a common reservoir that supplies water across a number of communities and with a structured management system offered by the government (e.g. Tono and Vea irrigation schemes).

In the second stage, simple random sampling was used to select 300 irrigated households from a sample frame of 1813 households. Thus, 50 irrigated households were randomly selected from each of the six communities studied, representing more than 20% of the total households in each of the selected communities. In the last stage, one farmer was selected from each sampled household, who had to give consent for participation in the study.

### Study methods and analytical framework

This paper applied a sequential mixed methods approach, where the qualitative study using key informant interviews and focus group discussions preceded the quantitative method using a semi-structured questionnaire (Additional file 1). The main advantage of using sequential mixed methods is that the results of the first method are fed into the second method, such that the research problem is holistically addressed from different viewpoints through systematic triangulations. In other words, the results of the qualitative study made it possible for us to identify areas that needed more detailed and quantitative information, which informed the type of questions asked in the semi-structured questionnaire.

Analytically, descriptive statistics were used to present the results of types of EBFMPs identified in state and privately managed irrigation schemes. To identify the number of EBFMPs used, data were collected on the farm practices employed by each farmer in irrigation farming and rain-fed farming. These practices were then grouped into EBFMPs and non-EBFMPs. The contingent valuation method (CVM) was used to estimate farmers’ WTP through an iterative bidding technique for the sustainability of the EBFMPs. The amount a farmer was willing to pay to sustain the EBFMPs was obtained by finding the average value of the minimum and maximum amounts he/she was willing to pay (check Additional file 1 for details). This was to ensure that farmers did not overvalue or undervalue the services provided by the EBFMPs. To determine the factors that influence farmers’ WTP for EBFMPs sustainability, both the Chi-square automatic interaction detector (CHAID) and multiple linear regression analysis were employed. According to Amusa et al. [32], farmers’ characteristics such as age, education, farming experience, farm size and household size significantly influenced their WTP for agronomic soil conservation practices. Bani [36], using the multiple regression model, showed that age, gender, educational status, access to land and farmers’ perception of climate change are significant determinants of farmers’ WTP for the provision of environmental services. In addition, Aydogdu and Bilgic [33] reported that factors such as marital status, education, land ownership, use of modern technologies and perception of natural resources significantly predicted farmers’ WTP for efficient irrigation for sustainable farming.

From Greene [37], the multiple linear regression model is presented mathematically as follows:

1 $$y_{i} = x_{i1} \beta_{1} + x_{i2} \beta_{2} + \cdots + x_{ik} \beta_{k} + \varepsilon_{i}$$

where $$y = dependent\;variable$$, $$x_{1} ,x_{2} , \ldots , x_{k} = explanatory\;variables$$, $$\varepsilon = disturbance\;term.$$

Following previous studies [e.g. 32, 33, 36], the relationship between WTP for EBFMPs and the characteristics of farmers can be presented empirically as follows:

2 $$\begin{aligned} WTP_{i} & = \beta_{0i} + \beta_{1i} Age_{1i} + \alpha_{2i} Primary_{2i} + \alpha_{3i} JHS_{3i} + \alpha_{4i} SHS\_n\_above_{4i} \\ & \quad + \beta_{3i} Marital\_s_{3i} + \beta_{4i} EBFMPs\_k_{4i} + \beta_{5i} Soil\_p_{5i} + \beta_{6i} Farm\_size_{6i} \\ & \quad + \beta_{7i} Irrig\_type_{7i} + \varepsilon_{ki} \\ \end{aligned}$$

The variables modelled are described in Table 3. To determine the appropriate variables for the model, bivariate regressions were first conducted to test for association among selected variables from literature and WTP for EBFMPs. Thereafter, only the variables found to be significantly associated with WTP for EBFMPs were included in the model. The variables found not to be significantly associated with WTP for EBFMPs in the bivariate analysis are respondent’s sex and household size.

**Table 3:** Definition of variables and *apriori* expectations for the multiple regression

Variable	Variable definition	Units of measurement	Expected sign
WTP	Willingness to pay for EBFMPs	Cedi value	
Age	Age of farmer	Years	±
No education	Reference variable		
Primary	Primary education	Dummy (1 = had primary education, 0 = otherwise)	+
JHS	Junior High School (JHS) education	Dummy (1 = JHS, 0 = otherwise)	+
SHS_n_above	Senior High School (SHS) education and higher	Dummy (SHS and higher = 1, 0 = otherwise)	+
Marital_s	Marital status of the farmer	Dummy (1 = married, 0 = not in a union)	−
EBFMPs_k	Perceived knowledge of EBFMPs	Indexed on each EBFMP importance stated	+
Soil_p	Perception of soil fertility	Dummy (1 = fertile, 0 = less fertile)	−
Farm_size	Irrigable farm size	Acres	+
Irrig_type	Category of irrigation scheme	Dummy (1 = PIS, 0 = SIS)	+

The CHAID was used to give a preliminary tree diagnosis of farmers’ WTP for EBFMPs sustainability. CHAID analysis is an algorithm mostly employed to establish relationships between categorical response variable and other categorical predictor variables [38, 39]. The CHAID tree was used because it gave a visual explanation of the relationship between the dependent variable (WTP amount) and predictors [38, 39]. It also visualised the relationship between the explanatory variables. Besides, multiple linear regression was used to estimate the factors that determined the WTP for EBFMPs by farmers.

## Supplementary information

**Additional file 1.** Survey guide. Paper questionnaire used for data collection.

## Data Availability

The dataset for the study is available from the corresponding author upon request.

## Abbreviations

CGIAR: Consultative Group on International Agriculture Research
CHAID: Chi-square automatic interaction detector
PIS: privately managed irrigation schemes
CVM: Contingent Valuation Method
EBFMPs: ecosystem-based farm management practices
ECOWAP: ECOWAS Agricultural Policy
GSS: Ghana Statistical Service
SHS: Senior High School
SIS: state-managed irrigation schemes
MoFA: Ministry of Food and Agriculture
IWMI: International Water Management Institute
JHS: Junior High School
WIAD: Women in Agriculture Development
WLE: Water, Land and Ecosystems
WTP: willingness to pay
UDS: University for Development Studies

## Footnotes

1 Compost is an organic matter that has been decomposed and usually used to fertilize agricultural lands. It also helps to improve nutrition. Organic manure/compost usually provides energy for microbes and other minerals for the growth of crops.

2 Tillage is an activity of loosening the compactness of soil particles for agricultural production. Conservative tilling helps conserve soil carbon which maintains agricultural soil structure and fertility. It also helps to control soil erosion and siltation.

3 Conserving vegetation helps to regulate temperature levels in arable lands and maintain soil moisture. Fell leaves of trees prevent erosion and equally decompose to fertilize the soil. It also prevents salination.

4 A mulch is a layer of materials (most often leaves) applied to the surface of an area of soil to conserve moisture, reduce weeds growth and improve soil fertility.

5 Crop rotation is a system of farming that allows the variation in choice of crops to cultivate every year. It allows biological balancing of soil nutrients such as fixation of nitrogen and helps to control soil-borne diseases.

6 Intercropping with legumes is a system of farming where leguminous crops are cultivated with other crops. Legumes help to fix atmospheric nitrogen through their symbiotic relationship with *Rhizobium* spp.

7 An efficient drainage system ensures continuous water supply, prevents siltation and promotes healthy aquatic life.

8 Bunding is farm-based practice adopted by farmers to retain water and prevent loss of soil fertility within farmlands. Usually, farmers use either soil or stones in building bunds (elevated boundaries). There is higher implementation cost if there is shortage of stones.

9 An exchange rate of USD1 to GHS 4.76 is used for the paper (https://www.xe.com/currencyconverter/convert/?Amount=1&From=USD&To=GHS). This rate was accessed on September 9, 2018.
