# Supplementary material for: Enhancing healthy ecosystems in northern Ghana through eco-friendly farm-based practices: insights from irrigation scheme-types
Source: BMC Ecol. 2019 Sep 12;19:38. doi: 10.1186/s12898-019-0254-8 (PMC6740034; doi:10.1186/s12898-019-0254-8)
Supplement: Supplementary file 1 — Additional file 1. Survey guide. Paper questionnaire used for data collection. [file 12898_2019_254_MOESM1_ESM.docx]

**Survey Guide**

**Disclaimer and Consent:**

Dear Research Participant(s),

The aim of this survey is to assess the ecosystem-based management farm practices used by farmers in government and community managed irrigation schemes in the Kassena-Nankana Area. It is a study in partial fulfillment for the award of Master of Philosophy (M.Phil.) degree in Agricultural Economics at the University for Development Studies (UDS), Tamale. Thus, the information obtained through this interview is for academic purposes only and will be accorded the highest degree of confidentiality. Your consent is therefore sought to provide frank responses to the questions contained in this guide. Thank you for your cooperation and understanding.

Date of Interview: [ ][ ]/[ ][ ]/[ ][ ][ ][ ] Respondent's code: [ ][ ][ ]/[ ][ ][ ]/[ ][ ][ ]

| Government managed irrigation scheme- **GIS** | Community managed irrigation scheme- **CIS** |
| --- | --- |
| Biu irrigation scheme- **BIU** | Saboro irrigation scheme **– SAB** |
| Bonia irrigation scheme –**BON** | Pungu-Talenia irrigation scheme **- PUN** |
| Korania irrigation scheme- **KOR** | Paga irrigation scheme –**PGA** |

**SECTION A**

| **1.0 Socio- demographic data of farmer** | | | | **Code** | **Variable** |
| --- | --- | --- | --- | --- | --- |
| 1.1 Are you the household head? | 0-No, 1-Yes | | | [ ] | HH |
| 1.2 Indicate the composition of your household ***( people eating from the same pot)*** | **Household Category by Age** | **Total number of Household members** | | | HHsize |
|  |  | **Male** | **Female** | |  |
|  | 0-14 |  |  | |  |
|  | 15-24 |  |  | |  |
|  | 25-54 |  |  | |  |
|  | 55-64 |  |  | |  |
|  | 65+ |  |  | |  |
|  | **Total** |  |  | |  |
| 1.3 What is your major occupation? | 1- Crop farming  2-Trading or craftsmanship  3-Salary worker (state actual occupation) [______________________________] | | | [ ] | Occuptn. |
| 1.4 Sex of respondent | 0-Male 1-Female, | | | [ ] | Sex |
| 1.5 Age of respondent | Quote the exact age | | | [ ][ ] | Age |
| 1.6 Educational level | 1-No formal education  2- Primary education  3- JHS education  4- Vocational sch. /SHS/technical institute  5-Teacher/Agric/Nursing training colleges  6-Polytechnics/University | | | [ ] | Educ. |
| 1.7 Marital Status | 1- Single 2-Married  3-Seperated 4-Widowed | | | [ ] | Mar_sta |

**SECTION B**

| **2.0 The ecosystem-based farm management practices (EBFMPs) adopted by the farmer** | | |
| --- | --- | --- |
| 2.1 What type of crops do you cultivate? | **Irrigated farm(s)** | **Rain-fed farm(s)** |
|  | 1.____________________________  2.____________________________  3.____________________________  4.____________________________  5_____________________________  6_____________________________ | 1.____________________________  2.____________________________  3.____________________________  4.____________________________  5_____________________________  6_____________________________ |
| 2.2 What sustainable farm practices do you use on your farm(s)? | **Irrigated farm(s)** | **Rain-fed farm(s)** |
|  | *Tick [****√****] those adopted*  1-Manure/compost application [ ]  2-Conservative tillage /no tillage [ ]  3-Intercropping with legumes [ ]  4-Crop rotation [ ]  5-Mulching [ ]  6-Conservation of vegetation or trees  [ ]  7-Efficient drainage system [ ]  8-Soil/stone bunding [ ]  Others: .................................................................................................................................................................................... | *Tick [****√****] those adopted*  1-Manure/compost application [ ]  2-Conservative tillage /no tillage [ ]  3-Intercropping with legumes [ ]  4-Crop rotation [ ]  5-Mulching [ ]  6-Conservation of vegetation or trees  [ ]  7-Efficient drainage system [ ]  8-Soil/stone bunding [ ]  Others: .................................................................................................................................................................................... |
| 2.3 What are the benefits of using each of the above sustainable farm practices? | **______________________________________________________________** | |
|  | **__________________________________________________________________________________________________________________________________________________________________________________________**  ______________________________________________________________ | |
|  | :__________________________________________________________________________________________________________________________________________________________________________________________  __________________________________________________________________________________________________________________________________________________________________________________________  ____________________________________________________________________________________________________________________________  ____________________________________________________________________________________________________________________________  ____________________________________________________________________________________________________________________________ | |
| 2.4 Why have you not used all the sustainable farm practices? |  | |
| 2.5 What other farming practices have you used that are not sustainable? | **Irrigated farm(s)** | **Rain fed farm(s)** |
|  | *Tick [****√****] those adopted*  1- Excessive fertilizer  application [ ]  2-Excessive insecticides and  herbicides use [ ]  3-Cutting of trees [ ]  4-Leaking canals and general poor  water management [ ]  5-Bush burning [ ]  6-Excessive tillage [ ]  7-Others (specify)  [____________________________] | *Tick [****√****] those adopted*  1- Excessive fertilizer  application [ ]  2-Excessive insecticides and  herbicides use [ ]  3-Cutting of trees [ ]  4-Leaking canals and general poor  water management [ ]  5-Bush burning [ ]  6-Excessive tillage [ ]  7-Others (specify)  [____________________________] |
| 2.6 What are the reasons for using the above unsustainable farm practices? | **_____________________________**  **______________________________**  **______________________________** | **_____________________________**  **______________________________**  **____________________________** |
| 2.7 What do you think are the effects of the above unsustainable farm practices? | **_____________________________**  **______________________________**  **______________________________**  **______________________________** | **_____________________________**  **______________________________**  **______________________________**  **______________________________** |
| 2.8 What is your perception about the soil fertility | 1. Fertile [ ]  0. Not fertile [ ] | 1. Fertile [ ]  0. Not fertile [ ] |

**SECTION C**

| **3.0 Factors influencing the adoption of ecosystem-based farm management practices.** | | | | | |
| --- | --- | --- | --- | --- | --- |
|  |  | | **Code** | **Variables** | |
| 3.1 Indicate the number of acres of your farmland(s) under cultivation. | **Irrigated farm(s)** | | [ ][ ] | Farm_size | |
|  | **Rain-fed farm (s)** | | [ ][ ] |  |  |
| 3.2 Indicate your level of ownership of the farmland(s) under cultivation. | **Irrigated farm(s)** | 1- Full ownership  2- Share ownership  3-Rented  4-Gift | [ ] | Land_ownsp | |
|  | **Rain-fed**  **farm (s)** | 1- Full ownership  2- Share ownership  3-Rented  4-Gift | [ ] |  |  |
| 3.3 Do you have access to that particular land under cultivation in ***3.1***?  ***( skip if respondent have full ownership of land)*** | **Irrigated farm(s)** | 0-No  1-Yes | [ ] | Land_access | |
|  | **Rain-fed**  **farm (s)** | 0-No  1-Yes | [ ] |  |  |
| 3.4 Indicate the distance of your farmland under cultivation from your home. | **Irrigated farm(s)** | *Write the approximated distance in meters/kilometers* |  | Farm_distance | |
|  | **Rain-fed**  **farm (s)** | *Write the approximated distance in meters/kilometers* |  |  |  |
| 3.5 Is there farmer(s) who have adopted sustainable farm practices close to your farmland(s)? | **Irrigated farm(s)** | 0-No  1-Yes | **[ ]** | Close_adopters | |
|  | **Rain-fed farm (s)** | 0-No  1-Yes | **[ ]** |  |  |
| 3.6 How many times do you get extension officer visiting your farm in a year? | Quote the exact number of times | | **[ ][ ]** | Ext.Visits | |
| 3.7 Is there any farmers' association that you belong? | 0-No 1-Yes  ***If yes, name?*** [______________________________] | | **[ ]** | Grp_Mem | |
| 3.8 Do you have access to organic manure or compost? | 0-No  1-Yes | | **[ ]** | Access_ Org | |
| 3.9 How many years have you been farming? | **Irrigated farm(s)** | | [ ][ ] | | Yrs_Experience |
|  | **Rain-fed farm (s)** | | [ ][ ] | |  |
| 3.10 Are you into livestock rearing? | 0-No  1-Yes | | | | Livestock_ownshp |

**SECTION D**

| **INSTRUCTIONS**: Diligence should be taken to explain the importance of the EBFMPs to farmers with the aid of the ***reference guide*** attached to the questionnaire before commencement of this session. | | | | | | | | |
| --- | --- | --- | --- | --- | --- | --- | --- | --- |
| **4.0 Farmer's Willingness to Pay (WTP) value for the sustainability of the EBFMPs** | | | | | | | | |
|  |  | | | | | **Code** | **Variable** | |
| 4.1 Do you usually get very good yield with the use of fertilizer? | 0-No  1-Yes | | | | | [ ] |  | |
| 4.2 What are some of the problems you are currently experiencing from the use of fertilizer? | ___________________________________________________________  ___________________________________________________________ | | | | | | | |
| 4.3 What can be done to salvage the situation? | ___________________________________________________________  ___________________________________________________________ | | | | | | | |
| 4.4 How much do you spend averagely to fertilize the soil and control pests and diseases in a season? |  | **Qty** | **Unit** | **Unit price** | **Amt GH₵** | **[ ]** | |  |
|  | Fertilizer |  |  |  |  |  |  |  |
|  | Pesticides |  |  |  |  |  |  |  |
|  | Herbicides |  |  |  |  |  |  |  |
| 4.5 Considering the cost you usually incur on fertilizer in **4.4**, how much will you be ***willing to pay*** for **compost** to fertilize your soil per an acre? | *Probe for the maximum s/he will be WTP*  1- GH₵ 0 [ ]  2- GH₵ 1-50 [ ]  3- GH₵51-100 [ ]  4- GH₵101-150 [ ]  5- GH₵151-200 [ ]  6- GH₵ 201-250 [ ]  7- GH₵ 251-300 [ ]  8- GH₵ 301-350 [ ]  9- GH₵ 351-400 [ ]  10- GH₵401-450 [ ]  11- GH₵451-500 [ ]  12- above GH₵500 [ ] | | | | | **[ ]** | |  |
| 4.6 How much will you also be ***willing to pay*** the environment for controlling ***diseases and pests*** considering the cost on Pesticides per an acre in **4.4?** | *Probe for the maximum s/ he will be WTP*  1- GH₵ 0 [ ]  2- GH₵ 1-50 [ ]  3- GH₵51-100 [ ]  4- GH₵101-150 [ ]  5- GH₵151-200 [ ]  6- above GH₵ 200 [ ] | | | | | **[ ]** | |  |
| 4.7 How much will you be ***willing to pay*** the natural environment for maintaining the ***temperature level of crops*** on your farm(s)? | *Probe for the maximum s/he will be WTP*  1- GH₵ 0 [ ]  2- GH₵ 1-50 [ ]  3- GH₵51-100 [ ]  4- GH₵101-150 [ ]  5- GH₵151-200 [ ]  6- GH₵ 201-250 [ ]  7- above GH₵250.00 [ ] | | | | | **[ ]** | |  |
| 4.8 What usually happens to your crops and/or economic life when there is shortage of water for farm business? | _____________________________________________________________  _____________________________________________________________  _____________________________________________________________ | | | | | | | |
| 4.9 Considering the effects in **4.8**, how much are you ***willing to pay*** for the services provided by the sustainable farm practices in ensuring regular water supply? | *Probe for the maximum s/he will be WTP*  1- GH₵ 0 [ ]  2- GH₵ 1-50 [ ]  3- GH₵51-100 [ ]  4- GH₵101-150 [ ]  5- GH₵151-200 [ ]  6- GH₵ 201-250 [ ]  7- above GH₵250.00 [ ] | | | | | **[ ]** | |  |

**SECTION E**

| **7.0 How farmers' livelihood conditions have been affected** | | | | | | | | | | | | |
| --- | --- | --- | --- | --- | --- | --- | --- | --- | --- | --- | --- | --- |
|  | | | | | | | | | | | | |
| **A. Food availability situation in farmer's household for the past 12 months** | | | | | | | | | | | | |
| Instructions: tick[ √] and score the appropriate condition for each month | | | | | | | | | | | | |
| **Grading** | Sufficient =3, Insufficient=2, Extreme shortage=1 | | | | | | | | | | | |
| **Month** | Jan | Feb | Mar | Apri | May | Jun | Jul | Aug | Sep | Oct | Nov | Dec |
| **Score** |  |  |  |  |  |  |  |  |  |  |  |  |
| **Total Score** |  | | | | | | | | | | | |

| **B. Housing condition** | | | |
| --- | --- | --- | --- |
| *Instructions: tick[ √] the appropriate condition for each housing condition* | | | |
|  | | Score | Total Score |
| Ownership of house | Personal house= 3 Family house= 2 Rented =1 | [ ] | [ ] |
| Number of rooms roofed with roofing sheets (e.g. zinc or aluminium sheets etc.). | All rooms =3 Half of the rooms =2 Below half=1 | [ ] |  |
| Number of rooms built with cement blocks. | All rooms = 3 Half of the rooms= 2 Below half=1 | [ ] |  |
| Number of rooms floored. | All rooms = 3 Half of the rooms= 2 Below half=1 | [ ] |  |
| General impression. | Excellent =4 Very good =3 Good= 2 Bad= 1 | [ ] |  |

| **C. Health situation** | | | |
| --- | --- | --- | --- |
| ***Availability of health facility*** | | Score | Total Score |
| Any close-by health facility? | District hospital/ Community health centre/ CHPS compound = 2  No health post closely available =1 | [ ] | [ ] |
| ***Access to health treatment*** | | Score | Total Score |
| District hospital/Community health centre | All household members= 3, Some members =2 , No access=1 | [ ] | [ ] |
| Pharmacy | Always =3 Difficult=2 Not at all=1 | [ ] |  |
| Herbal treatment | Always =3 Difficult=2 Not at all=1 | [ ] |  |

| **D. Water facilities** | | | | | | | | | | | | | | |
| --- | --- | --- | --- | --- | --- | --- | --- | --- | --- | --- | --- | --- | --- | --- |
| ***(i) Water source*** | | | | | | | | | | Score | | Total score | | |
| Source of water | Borehole/pipe= 4 Mechanized well=3 Uncovered well=2 River/pond=1 | | | | | | | | | [ ] | | [ ] | | |
| ***(ii) Water quality*** | | | | | | | | | | score | | Total score | | |
| Drinking water | Good= 3 Clean but smells or hard =2 Unclean=1 | | | | | | | | | [ ] | | [ ] | | |
| ***(iii) Availability of water*** | | | | | | | | | | | | | | |
| **Grading** | Adequate =3 Inadequate=2 Scarcity =1 | | | | | | | | | | | | | |
| **Month** | Jan | Feb | Mar | Apr | May | Jun | Jul | Aug | Sep | | Oct | | Nov | Dec |
| **Score** |  |  |  |  |  |  |  |  |  | |  | |  |  |
| **Total Score** |  | | | | | | | | | | | | | |

| **E. Freedom in cash expenditure** | | | | | | |
| --- | --- | --- | --- | --- | --- | --- |
| **Things for expenditure** | Level of decision in cash expenditure | | | | Score | Total score |
|  | Farmer =4 | Farmer and spouse=3 | Spouse =2 | Extended family =1 |  |  |
| Daily expenditure |  |  |  |  | [ ] | [ ] |
| Investment on land |  |  |  |  | [ ] |  |
| Children education |  |  |  |  | [ ] |  |
| Health |  |  |  |  | [ ] |  |
| Household assets |  |  |  |  | [ ] |  |

| **F. Sanitation** | | **Score** | **Total score** |
| --- | --- | --- | --- |
| Access to a toilet facility | Owned toilet=3 Community toilet=2 None=1 | [ ] | [ ] |
| Condition of toilet | Hygienic =3 Better= 2 Unhygienic =1 | [ ] | [ ] |

| **G. Participation in social activities** | | **Score** | **Total score** | |
| --- | --- | --- | --- | --- |
| Participation level | -Freedom to participate in any gathering=2  -Limited freedom to participate=1 | [ ] | [ ] |  |

| **H. Health of ecosystem services** | | | | **Score** | **Total score** |
| --- | --- | --- | --- | --- | --- |
| Ecosystem services | Sustained =3 | Deteriorating =2 | Worsened =1 |  | [ ] |
| Fish availability |  |  |  | [ ] |  |
| Vegetation for animals |  |  |  | [ ] |  |
| Availability of medicinal plants |  |  |  | [ ] |  |
| Availability of fuel wood |  |  |  | [ ] |  |
| Fruits availability |  |  |  | [ ] |  |
| Water availability for recreational purposes |  |  |  | [ ] |  |
| Flood control |  |  |  | [ ] |  |
| Erosion control |  |  |  | [ ] |  |
| Siltation control |  |  |  | [ ] |  |
| Pests and diseases control |  |  |  | [ ] |  |

**SECTION F**

**Factors that might affect farmers' livelihood**

| **Crop farm income of household members for the past 12 months** | | | | | | |
| --- | --- | --- | --- | --- | --- | --- |
| **Household Members** | | **Crop** | **(a)Total output of crop** | **(b) Units {e.g. bags, bowls etc}** | **(c) Crop Value per unit GH₵** | **(d) Total crop value {a*c} GH₵** |
| **Household Head** | **Irrigated** |  |  |  |  |  |
|  |  |  |  |  |  |  |
|  |  |  |  |  |  |  |
|  |  |  |  |  |  |  |
|  | **Rain-fed** |  |  |  |  |  |
|  |  |  |  |  |  |  |
|  |  |  |  |  |  |  |
| **Spouse** | **Irrigated** |  |  |  |  |  |
|  |  |  |  |  |  |  |
|  |  |  |  |  |  |  |
|  | **Rain-fed** |  |  |  |  |  |
|  |  |  |  |  |  |  |
| **Children and others** | **Irrigated** |  |  |  |  |  |
|  |  |  |  |  |  |  |
|  | **Rain-fed** |  |  |  |  |  |
|  |  |  |  |  |  |  |
| **TOTAL** | |  | | | |  |

| **Inventory of Farmer's household livestock *(Skip if respondent does not rear livestock)*** | | | | | |
| --- | --- | --- | --- | --- | --- |
| **Type of livestock** | **Number sold over the last 12 months** | **Number dead** | **Number stolen** | **Number killed for household consumption** | **Number in stock** |
| Cattle |  |  |  |  |  |
| Sheep |  |  |  |  |  |
| Goat |  |  |  |  |  |
| Pigs |  |  |  |  |  |
| Poultry birds |  |  |  |  |  |
|  |  |  |  |  |  |
|  |  |  |  |  |  |
| **Total** |  |  |  |  |  |

| **Other livelihood supporting income of farmer's household ( Skip if not applicable )** | | |
| --- | --- | --- |
| Remittances received | Value in Gh₵ | [ ] |
| Average monthly salary of Household head **( *if a salary earner*)** | Value in Gh₵ | [ ] |
| Average monthly salary of Household head's spouse **(*if a salary earner*)** | Value in Gh₵ | [ ] |
| Others  [ ______________________________] |  |  |

Name of interviewer: [__________________________________] Signature: [____________]

Contact of interviewee (if any): [________________________________________________]
